# Common Salt in Intermittent Fever

**Published:** 1853

**Authors:** Joseph C. Hutchison

**Affiliations:** Brooklyn, N. Y.


*Common Salt in Intermittent Fever.*—By Joseph C. Hutchison, M. D., of Brooklyn, N. Y.

Notwithstanding the favorable reports which have been made, the remedy has been but little used in this country, because its antiperiodic influence was doubted. And it has been thought not inappropriate again to introduce the subject to the notice of the profession, with the hope that it may receive more general attention. The experiments were commenced for the purpose of determining, with some degree of accuracy, a knowledge of its value in intermittent fever, by exhibiting it in a large number of cases. And no better field could have been selected for the experiment, not even the far-famed Pontine marshes, whether we consider the very general prevalence of the disease, or the protean and obstinate forms it here sometime assumes. In consequence, however, of my removal from this city, it was prescribed in but twenty-two cases, occurring in twenty individuals, a summary of which is now presented as they were recorded; there has, consequently, been no attempt at a selection of the cases. I was enabled to ascertain the permanency of the cures in but three patients, and the condition of the spleen in but one. It would have been very desirable to have observed its effect in the spleen, which, according to Piorry, is diminished in volume with marvellous celerity. These unavoidable imperfections in the history of the cases arise from the fact, that most of the patients resided at a considerable distance from my office, and were prescribed for without having been examined; and, indeed, from the very general prevalence of the disease, most persons have become so familiar with the use of quinia (which is usualy kept in the house, and prescribed as a matter of economy and convenience by the head of the family, when necessary), that a resort to the physician in ordinary cases is rarely thought of. But I think we may safely surmise, that hypertrophy of the spleen existed in every case, excepting, perhaps, in cases 13 and 16, which were recent cases, oocurring in very young subjects; because, in malarious districts, this pathological condition exists almost universally, and can be readily detected by careful percussion, even in persons who have never had what are commonly termed miasmatic diseases (intermittent and remittent bilious fevers), and who enjoy apparent good health. It appears to be the direct effect of the malarial poison, and it is a fact well known to practitioners in miasmatic districts, that in all diseases occurring there, even in those not strictly of malarial origin, the morbid effects of this poison are more or less obvious, requiring for their treatment specific remedies, in addition to their ordinary therapeia.

Case 1.—Elisha E., æt. 3 years, prescribed for April 28th, 1852. He had intermittent fever last spring, which was then checked, but returned the ensuing autumn, and has recurred at irregular intervals to the present time. Quinia, arsenic, &c., have been used. The present attack is of the tertian type. Skin pallid and sallow, and his general health is somewhat impaired. ℞. Chloridi sodii ʒij; mucil. ulmi f℥j. M. A teaspoonful to be taken every three hours, commencing twenty-four hours before the next paroxysm is expected.

I was informed by his father to-day (May 3d), that the paroxysms were at once arrested by the above mixture. There was no disturbance of the stomach or bowels. Thirst.

Case 2.—S. M., farmer, æt. 30, prescribed for May 3d,

1852. He contracted intermittent fever last autumn, and relapses have occurred, at intervals of a few weeks, to the present time, notwithstanding the abundant use of quinia, and other tonics and antiperiodics. The present attack has existed for several days, and presents the quotidian form. His complexion exhibits the appearance characteristic of the disease. ℞. Chl. sodii ℥j; mucil. ulmi. f℥ iv. M. Tablespoonful every two hours during the next intermission, as there is not now sufficient time to take the requisite quantity before the paroxysm is expected. On the 5th the chill returned at the usual time (although the salt was taken as directed), but in a much milder form, thirst; four dejections without pain, and no nausea.

This patient was accustomed to having his chills arrested immediately by sulphate of quinia, and was unwilling to risk again the prophylactic virtues of the chloride. But, from the marked diminution of the violence of the paroxysms, there is great probability that, if it had been continued, it would have arrested the disease. The disorder was suspended by liq. potass. arsenit. gtt. xv., ter. in die.

Case 3.—Elisabeth E, æt. 13, quotidian intermittent; prescribed for May 5th. The present attack has existed several days. She had the disease last autumn, and occasional relapses have occured to the present time, sometimes assuming the quotidian form, and at others the tertian. Has been accustomed to use quinia to arrest the paroxysms. General health pretty good during the intervals of the attacks. She was ordered chl. sodii ʒij in mucilage every three hours until six drachms were taken, commencing eight hours before the return of the paroxysm was expected. The disease was thus immediately arrested. She vomited once after taking the second dose, which was soon repeated and well retained. Four dejections, without griping; no nausea; thirst.

She was directed to take a drachm of the chloride night and morning for three or four weeks, as a prophylactic. The disease subsequently returned, but I could not learn with accuracy how long it was kept in abeyance. Her father informed me, however, that it did not return so soon as it had formerly done, after the use of quinia.

CASE 4.—T. G. M., farmer, æt. 30, prescribed for the 5th of May, for the tertian form of intermittent fever. He had the disease last September ; it was then checked, and he has been free from it until three weeks since, when it returned, and was promptly arrested by sulph. quinia. Since then, he has been using, as a prophylactic, bitters composed of bark, gentian and rhubarb, but a relapse occurred, notwithstanding, on the 21st day. His skin is pallid, but he feels pretty well during the intermission. He was ordered chloride of sodium ℨij in mucilage, every four hours, commencing fifteen hours before the paroxysm is expected to return, and to take four doses. Only six drachms (three doses) were taken, the paroxysm having occurred one hour after the last dose, and six hours earlier than it was expected. There was thirst, but no disturbance of the stomach or bowels. This patient objected strongly to the taste of the remedy; and as he doubted its efficacy, and was very anxious to have the disorder *immediately* arrested, I prescribed sulph. quinia grs. x, aromatic powders grs. x, opium gr. j ; to be taken five hours before the next paroxysm was expected. This promptly checked the disease.

The above case was very unsatisfactory in determining the value of the remedy. It cannot be counted a failure, nor, indeed, have we any positive evidence it would have produced the effect desired, if the quantity prescribed had been exhibited. I may remark, *en passant*, that I usually prescribe ten grains of quinia at a single dose, in intermittent fever, five hours before the accession of the paroxysm, combined with an equal quantity of aromatic powder, when there is a want of gastric sensibility, and sometimes with an opiate, or a purgative dose of calomel, when indicated. Given in this dose, a concentrated effect is developed at the proper time, which makes it more potent in arresting the disease, than if distributed over a long interval: a second dose is rarely necessary. It

11

is preferable, also, as a matter of convenience to the patient.

Case 5.—Leonard W., æt. six years, prescribed for May 8th, 1852. He contracted intermittent fever last November, and relapses have repeatedly occurred during the winter and the present spring. He has now had two paroxysms, occurring on alternate days. Skin pallid and sallow, but he complains of no particular disorder during the intermission. ℞. Chloridi sodii ʒijss. To be taken in mucilage, before the next paroxysm is expected. The chill returned on the 10th, at the usual time, and the following mixture was then ordered: ℞. Chloridi sodii ʒiv; mucil. ulmi fʒij.; tablespoonful every four hours, commencing fifteen hours before the next paroxysm is expected. This increased quantity of the salt promptly arrested the disease. No nausea or purging; thirst. From the readiness with which the second paroxysm was arrested by four drachms of salt, it seems not improbable that the first might have been prevented, if as large a quantity had been given. Of this, however, we can only surmise.

Case 6.—A negro women belonging to Mrs. S., æt. 20. She has been subject to the tertian type of intermittent fever for several months, it having been from time to time suspended by quinia. The present attack (May, 1852) has existed four or five days. Her general health is somewhat impaired: ℞. Chloridi sodii ʒix; mucil. ulmi fʒiij, M. A table-spoonful every three hours, commencing eighteen hours before the next paroxysm is expected. The above dose promptly arrested the disease, nor had there been a relapse up to May, 1853, (one year from the time the salt was prescribed), as I learned from Mrs. S. There was thirst, but no disturbance of the stomach or bowels followed the use of the remedy.

The promptness and permanency of the cure in this case is very remarkable, when we consider the length of time the patient had been subject to the disorder and the frequent relapses which occurred when it had been suspended by the use of quinia. So well pleased was Mrs.

S. with the effect of the remedy in this case, that she subsequently wrote to me from Virginia, where she had gone to visit her daughter, requeating the recipe for the "invaluable prescription," for the benefit of her friends there.

Case 7.—Ellen B., æt. four years prescribed for August 1st, 1852. She had intermittent fever for the first time last summer, and since then, the paroxysms have recurred at irregular intervals to the present time. They have been suspended sometimes by quinia, and at others by liq. potass. arsenit. The present attack has existed for several consecutive days. Skin pallid and icterode, and her general health is considerably impaired. ℞. Chloride sodii ʒiv; mucil. ulmi fʒij. M. A table-spoonful every three hours, commencing twelve hours before the next paroxysm is expected. The disease was thus at once arrested. Thirst; no disturbance of the chylopoietic viscera.

Case 8.—Mary B., æt. six, sister of last patient, was prescribed, at the same time, for a like form of disease. She has also been subject to occasional attacks of intermittent fever for the last twelve months, which have been checked from time to time by the usual remedies. Her aspect and general health is similar to that of the last patient. She was also ordered the same prescription, which had the effect of at once suspending the disease.

Case 9.—Anna B., æt. nine, sister of the last two patients, was prescribed for August 4th. She has also been a victim of intermittent fever for twelve months or more, the paroxysms recurring every few weeks. The present attack, which has existed several days, offers the quotidian variety. Skin pallid and icterode, and her general health is much impaired. ℞. Chloridi sodii ʒv; mucil. ulmi fʒiiss M. A table-spoonful every third hour, commencing fifteen hours anterior to the time for the next paroxysm. By this treatment, though the disease was not checked, the paroxysm was postponed two hours. The same plan of treatment was directed for the next day, that had been pursued the day previous, which completely arrested the disease.

We may very justly infer from the history of the last three patients, that there was considerable visceral derangement and impairment of the general health. The cases had proved exceedingly rebellious to the ordinary remedies, and even when under the potent influence of sulph. quinia, the paroxysms often persisted for several days *seriatim*. The antiperiodic effects of the chloride here was certainly very strongly marked. I regret that the permanency of the cure was not reported to me.

Case 10.—A negro girl, belonging to Mr. C., æt. 15, prescribed for August 5th. She had intermittent fever last autumn, and the ensuing spring, but has been free from it for several months, until to-day. Her general health is moderately good. As it was uncertain what type the disease would assume, she was ordered chl. sodii 3vj dissolved in mucilage, to be taken in divided doses at suitable intervals, before the hour for the chill to-morrow, anticipating it might prove to be of the quotidian type. The paroxysm returned on the next day, notwithstanding the treatment, and for two consecutive days without any obvious amelioration. Mrs. C. then gave a teaspoonful of corn meal mixed with water, every two hours during the next intermission, after which the disease did not return. There was thirst, but no disturbance of the stomach or bowels from the chloride.

Corn meal, I may remark, is a domestic remedy for ague and fever, often used by this family, and others in that neighborhood, and it is said, in most instances, to check it at once. It would *seem*, in the above case, to have been efficacious. At any rate, the salt was here considered a failure.

Case 11.—A negro girl, belonging to Mr. S., æt. twelve, prescribed for August 6th. She has had the tertian form of intermittent fever four or five days. Never had the disease before, although it has prevailed extensively in family. General health good. As the paroxysm was expected in nine hours from this time, she was ordered chl. sodii, ʒi, dissolved in mucilage, *every hour*, until she takes six doses. On the 7th, the disease returned at the usual time, each dose of the chloride having been soon followed by emesis. She was then directed ʒss doses at sufficient intervals to allow ʒvij to be taken during the next intermission. On the 11th, I learned that the vomiting continued, though less frequently, and that the paroxysms still recurred, but in a milder form. Quinia was then prescribed, and the disease immediately subsided. Thirst, but no nausea or dejections.

In consequence of the emetic effect of the salt in the above case, it cannot be considered a fair test of its antiperiodic virtue, as it is probable a very small portion of the amount taken was retained. The paroxysms, notwithstanding, became milder, but this may have been owing to the influence of the vomiting *per se.*

Case 12.—A negro girl, æt. 18, the property of Mr. D., prescribed for, August 20th. Her master informs me that she has had the tertian form of intermittent fever for three months, it having been suspended from time to time by quinia, &c., to return again on the 7th, 14th, or 21st days. She has now had two or three paroxysms, occurring on alternate days. Her general health is somewhat impaired: ℞. Chl. sodii ʒix; mucil. ulmi f℥iij. M. A tablespoonful every three hours, commencing eighteen hours before the next paroxysm is expected. Nine drachms of the salt were taken, which immediately arrested the disease; nor did it return until November 1st, as will be seen by referring to Case 20. No nausea or purging; thirst.

Case 13.—Child of Mr. D., æt. two years; prescribed for August 23d, 1852. It has had the quotidian form of intermittent fever six or seven days; never had the disease before: ℞. Chloride sodii ʒiss; mucilage ulmi f℥ss. M. A teaspoonful every three hours, commencing twelve hours before the time for the next paroxysm. This patient subsequently had several paroxysms; the chloride, as I

learned from its father, having produced no obvious benefit and no disturbing effects. It was not repeated. Sulph. quinia was then prescribed, which at once suspended the disease.

The parents of this child were of the lower order of Irish, who, as is well known to physicians that prescribe much for them, are not in the habit of rigidly following directions in the administration of medicines; and I have reason to suspect that the mixture was not exhibited properly in this instance.

Case 14.—Joseph M. æt. sixteen, prescribed for August 28th. He contracted intermittent fever last autumn, and since then he has suffered with frequent attacks of the disorder, which have been suspended by the preparations of bark, &c. The present attack, which commenced five days since, offers the tertian form. Skin pallid and icterode; but he feels very well during the intervals of the attacks. ℞. Chloride sodii ʒvi; mucilage ulmi f℥ij. M. A table-spoonful every three hours, commencing twelve hours before the next paroxysm is expected. He had a paroxysm at the usual time, on the day he took the above prescription, but none subsequently, although nothing more was taken to arrest the disease. Free vomiting of bilious matter occurred soon after the cold stage subsided.

Case 15.—Elizabeth M., æt. twelve, sister of last patient. She was also prescribed for on the 28th, for the same form of intermittent, which had existed with frequent suspensions since the preceding autumn. Skin pallid and sallow; general health impaired: ℞. Chloride sodii ʒvi; mucil. ulmi f℥ij. M. A table-spoonful every three hours, commencing twelve hours before the time for the next paroxysm.

This prescription had no obvious influence in mitigating the disorder, and was not repeated, because the patient lived some distance from me, and did not again apply until she had had four or five additional paroxysms. Quinia was then prescribed at her request, which at once checked the disease. Copious bilious vomiting also occurred in this case soon after the algid stage of the paroxysm which immediately succeeded the use of the chloride, had subsided.

Case 16.—Son of Mr. J. S., æt. two and a half years; prescribed for September 17th, 1852. He has had the quotidian form of intermittent fever for several days; has never had the disease before. Health always perfectly good anterior to the present indisposition. The following mixture was ordered: ℞. Chl. sodii ʒiss: mucil. ulmi f℥i. M. Two teaspoonfuls every three hours, commencing twelve hours before the period for the return of the paroxysm. The disease was thus at once arrested. No nausea, but some alvine discharges; thirst.

Case 17.—A sister of the last patient, æt. five years; prescribed for also on the 17th, for the same form of the disease. This, too, is a recent case, and the patient's general health is pretty good. The paroxysms have now recurred for five consecutive days: ℞. Chl. sodii ʒij; mucil. ulmi f℥ij; to be taken in table-spoonful doses every three hours, commencing twelve hours before the next paroxysm is expected. The direction was misunderstood, so that but half the quantity prescribed was administered, which, however, had the effect of promptly arresting the disease. Several dejections; no nausea or vomiting; thirst.

